# Image findings of cranial nerve pathology on [18F]-2- deoxy-D-glucose (FDG) positron emission tomography with computerized tomography (PET/CT): a pictorial essay

**DOI:** 10.1186/s40644-015-0054-0

**Published:** 2015-12-03

**Authors:** Osama A. Raslan, Razi Muzaffar, Vilaas Shetty, Medhat M. Osman

**Affiliations:** Department of Radiology, Division of Nuclear Medicine, St Louis University, 3635 Vista Avenue, Saint Louis, MO 63110 USA; Department of Radiology, Division of Neuroradiology, St Louis University, 3635 Vista Avenue, Saint Louis, MO 63110 USA

## Abstract

This article aims to increase awareness about the utility of ^18^F -FDG-PET/CT in the evaluation of cranial nerve (CN) pathology. We discuss the clinical implication of detecting perineural tumor spread, emphasize the primary and secondary ^18^F -FDG-PET/CT findings of CN pathology, and illustrate the individual ^18^F -FDG-PET/CT CN anatomy and pathology of 11 of the 12 CNs.

## Background

Conventional CT and MRI have been the imaging modalities of choice for evaluation of cranial nerve (CN) pathology. However, CN pathology can also be detected on [18 F]-2- deoxy-D-glucose (FDG) positron emission tomography with computerized tomography (PET/CT) imaging [1–3]. As FDG PET/CT is increasingly being used for oncologic imaging and more specifically for evaluation of head and neck (HN) cancer [4], PET/CT interpreters need to familiarize themselves with the image findings of CN involvement, which will greatly impact the staging and management of these patients.

Tumor related PET/CT findings include the perineural spread of HN tumors which represents a rare contiguous metastatic extension of tumor along a cranial nerve that portends to poor prognosis, even if the patient is asymptomatic [2, 5]. If present, treatment can be changed to include neck dissection, a larger radiation field, or adding adjuvant therapy [6–8]. Facial nerve involvement (CN VII) in parotid tumors may preclude facial nerve–sparing surgery or require additional treatment modality [9]. Patients with skin cancer and perineural invasion will require adjuvant radiation therapy even when clear margins are achieved with Mohs surgery [10, 11]. Also the degree of FDG uptake by the tumor as measured by the SUV max is an important prognostic marker for locally advanced nasopharyngeal cancer. High FDG uptake reflects more aggressive tumors that may require more aggressive treatment and carries a worse prognosis, as compared to the less aggressive low FDG tumors [12].

Non-tumor related benign and malignant cranial nerve pathology can also be incidentally detected during PET/CT oncologic imaging including schwannomas [13], optic nerve glioma [14], meningioma [15], and melanoma [15]. Gallium 68 (68Ga) 1,4,7,10-tetraazacyclododecane-1,4,7,10-tetraaceticacid (DOTA)–octreotate (DOTATATE, GaTate), has been shown to be more sensitive than FDG-PET/CT in detection of low grade somatostatin receptor positive tumors namely meningioma, esthesioneuroblastoma and schwannoma [16].

The purpose of this article is to describe the primary and secondary FDG-PET/CT findings of CN pathology and to provide a comprehensive illustration of the PET/CT cross-sectional anatomy and pathology of almost each individual CN, thus raising awareness and familiarity about incidental CN lesions seen on PET/CT, which will directly reflect on patient staging and management.

## Primary and secondary PET/CT findings of CN pathology

**Fig. 1:**
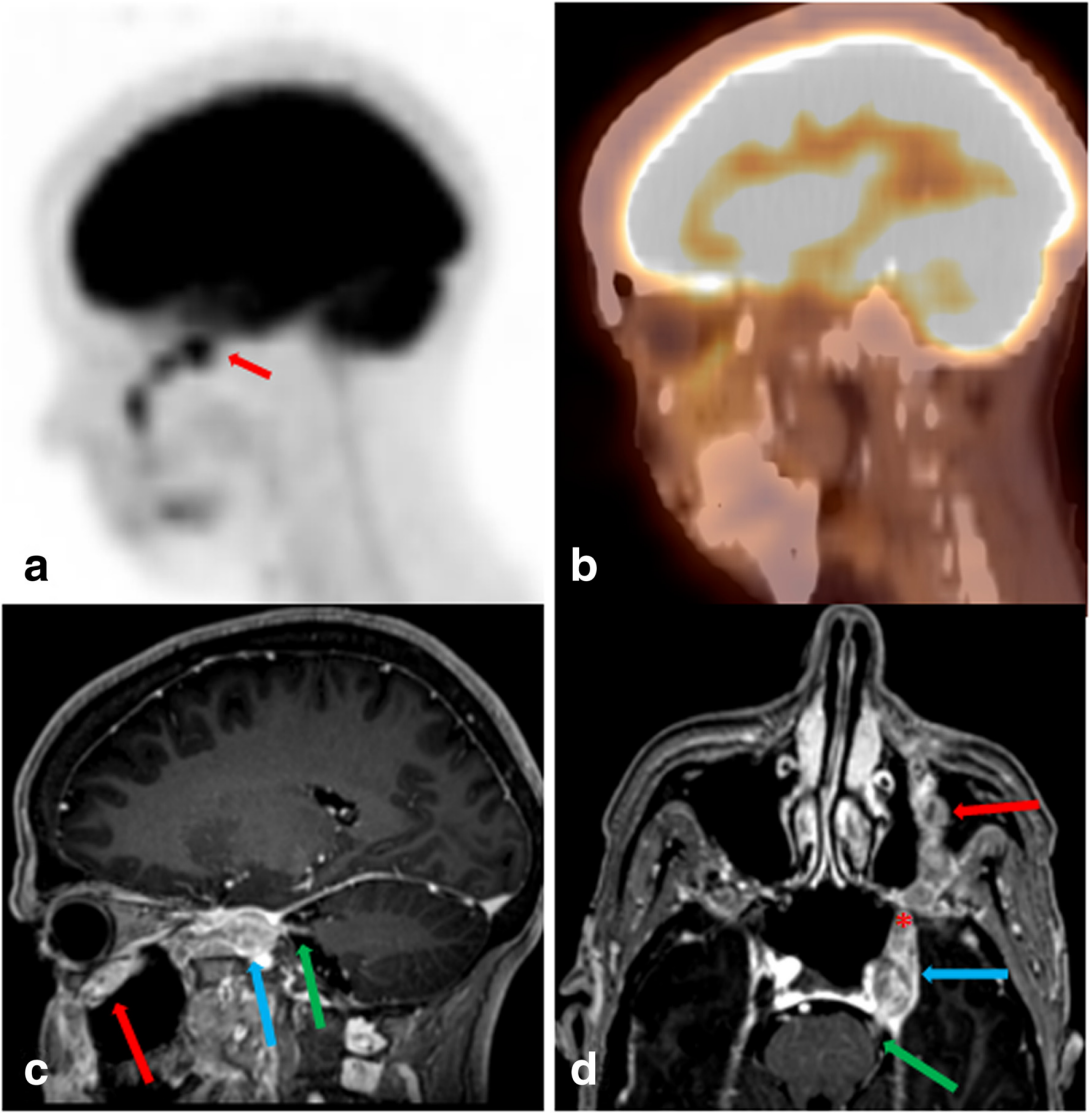
A 50-year-old female who is status post excision of left nasolabial melanoma,presenting with left perioral numbness. Sagittal unfused (**a**) and fused (**b**) FDG PET/CT shows alongitudinal hypermetabolic mass extending retrogradely along the expected course of theinfraorbital branch Of V2 in the orbital floor, with intracranial extension (arrow). Sagittal (**c**), axial (**d**) curved Multiplanar reformatted images (MPR) of fat suppressed post contrast T1 WI MRI confirming retrograde perineural metastatic melanoma, along the infraorbital branch of V2 (red arrow), through the foramen rotundum (asterix), into the cavernous sinus (blue arrow), to the cisternal portion of the left trigeminal nerve (green arrow)

The primary sign of CN pathology includes linear thickening or linear increased/decreased FDG activity along the expected course of the CN (Fig. 1). For this purpose, all three planes (axial, coronal and sagittal) and maximum intensity projection (MIP) images must be evaluated and correlations with all other available imaging modalities, e.g. (CT or MRI) which will often confirm the abnormality.

The secondary signs of CN pathology include widening or destruction at the corresponding skull base foramen, asymmetric atrophy or abnormal activity in the muscles supplied by the CN, or increased FDG activity related to synergistic/antagonistic muscle overcompensation to maintain function (Table 1).

**Table 1:** Clinical, primary and secondary findings of CN pathology

Symptoms/signs that trigger search pattern for CN pathology	Which CN to suspect?	What to look for and where to look for it on PET/CT?			
		Primary Sign	Secondary Signs		
		Abnormality along course of CN	Abnormal skull base foramen/ bone	Muscle atrophy	Over compensation
Anosmia	CN I (Olfactory)	Roof of nose and anterior cranial fossa	Cribriform plate of ethmoid	--	--
Visual loss	CN II (Optic)	Orbit, suprasellar cistern	Optic canal	--	--
Diplopia	CN III (Oculomotor)	Cavernous sinus	Superior orbital fissure (SOF)	Extraocular muscles (except superior oblique and lateral rectus muscles)	--
Vertical diplopia	CN IV (Trochlear)	Cavernous sinus	SOF	Superior oblique	--
Trigeminal Neuralgia	CN V (Trigeminal, main trunk)	Pons, prepontine cistern, Meckel's cave.	--	--	--
Paresthesia over forehead and eye	CN V1 (Ophthalmic division)	Cavernous sinus	SOF	--	--
Paresthesia over cheek	CN V2 (Maxillary division)	Cavernous sinus, cheek	Foramen rotundum, pterygopalatine fossa and infraorbital canal /foramen	--	--
Paresthesia over chin, trismus	CN V3 (Mandibular division)	Masticator space	Foramen ovale, mandibular canal and mental foramen	--	--
Lateral gaze diplopia	CN VI( Abducens)	Cavernous sinus, clivus	SOF	Lateral rectus	Ipsilateral Medial rectus
Facial palsy	CN VII (Facial)	Cerebellopontine angle , parotid space	Petrous bone, internal auditory canal (IAC), and stylomastoid foramen	--	--
Hearing loss/ imbalance	CN VIII (Vestibulocochlear)	Cerebellopontine angle	Petrous bone and IAC	--	--
Hoarseness	CN X (Vagus nerve, recurrent laryngeal branch)	Carotid space, tracheoesophageal grooves, around aortic root	Jugular foramen (JF)	Ipsilateral vocal cord	Contralateral vocal cord
Shoulder drooping	CN XI (Spinal accessory)	Carotid space	JF, foramen magnum	Sternomastoid and trapezius muscles	--
Dysarthria and dysphagia	CN XII (Hypoglossal nerve)	occipital condyles , Carotid space, base of tongue	JF, Hypoglossal canal	Ipsilateral hemitongue	Contralateral hemitounge

## Case presentation

### Olfactory nerve (CN I)

**Fig. 2:**
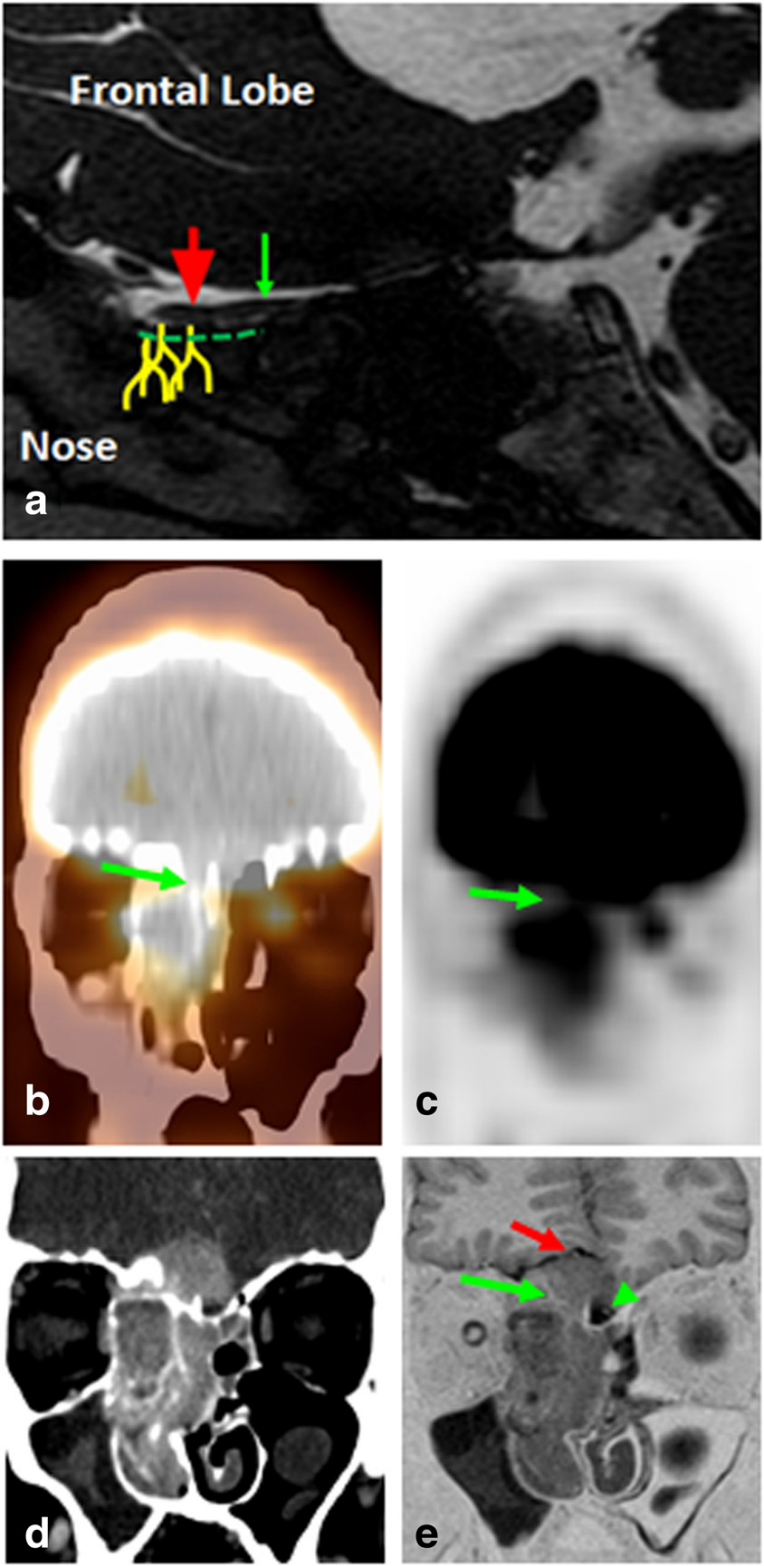
**a** Sagittal steady state free precession (SSFP) MRI image of the brain showing the olfactory nerve anatomy: The fibers in the superior nasal mucosa (yellow fibers), ascend through the fenestrated cribriform plate of the ethmoid bone (green dashed line) to reach the anteriorcranial fossa and continue as the olfactory bulb (red arrow) and tract (green arrow) coursing atthe inferior surface of the ipsilateral frontal lobe. **b**–**e** A 38-year-old male presented with a 6month history of anosmia and episodic epiphoresis, nasal stuffiness. Coronal fused (**a**) andunfused (**b**) PET/CT images demonstrate a large aggressive hypermetabolic mass centered on thesuperior aspect of the nasal cavity, extending superiorly into the right anterior cranial fossa andcribriform plate of ethmoid bone, involving the expected location of the right olfactorynerve/bulb/track (arrow). Our differential diagnosis was olfactory neuroblastoma (Esthesioneuroblastoma), or sinonasal carcinoma/melanoma. Coronal contrast enhanced CT (**c**) demonstrate the enhancing mass extending into the anterior cranial fossa at the expected locationof CNI. Coronal short tau inversion recovery (STIR) (**e**) images confirm the intracranial extension (arrow) with involvement of the right olfactory nerve/bulb (red arrow). Note the normal left olfactory track (green arrowhead). Surgical pathology showed an olfactory neuroblastom

Direct visualization of the CN I lesion is beyond the resolution of PET/CT, however CN I involvement should be suspected in lesions involving the superior sinonasal and anterior cranial fossa region. The differential considerations include olfactory neuroblastoma (Esthesioneuroblastoma), sinonasal carcinoma and melanoma (Fig. 2)

.

## Optic nerve (CN II)

**Fig. 3:**
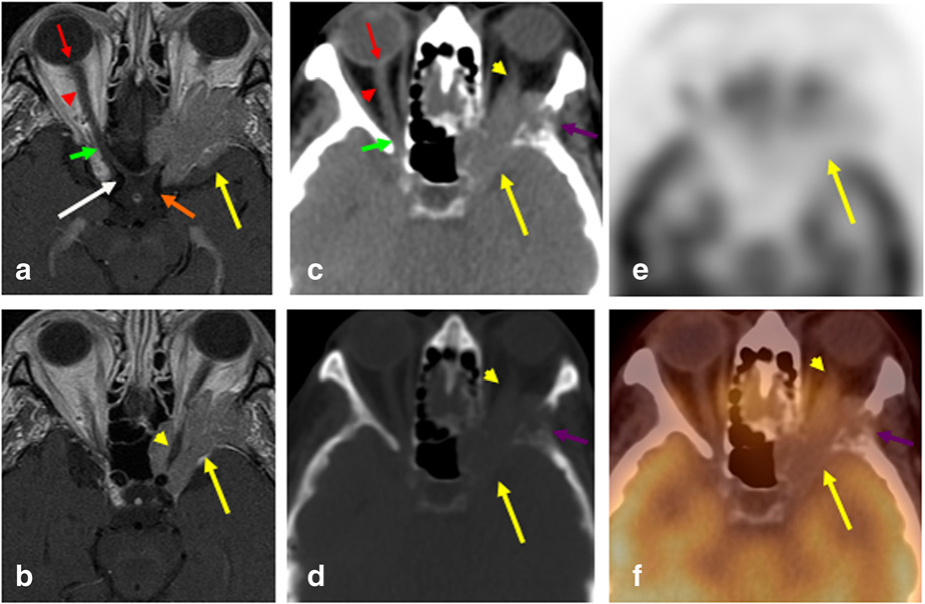
A 74-year-old female complaining of double vision. Axial post contrast T1 WI MRI (**a**, **b**), The right optic nerve demonstrates the normal anatomy, showing the four segments of the optic nerve, the retinal (red arrow), orbital (arrow head), canalicular (green arrow) and cisternal parts (white arrow). The optic chiasm is also seen (orange arrow). On the left side, ahomogenously enhancing extra axial mass is seen centered on the eroded left greater wing of thesphenoid bone encasing the left optic nerve. Axial CT soft tissue (**c**) and bone windows (**d**), axialPET (**e**), and fused PET/CT (**f**) images, showing a hypometabolic soft tissue mass causinghyperostosis and erosion of the left greater wing of the sphenoid and extending into the orbitalcavity along the optic nerve. Diagnosis sphenoid wing meningioma with intra orbital extension,encasing the optic nerve

The main differential considerations for CN II lesion include optic pathway glioma (OPG), optic nerve sheath meningioma, idiopathic orbital inflammatory pseudotumor, and optic neuritis. FDG activity in Optic nerve glioma is variable depending on its histological grade [17, 18]. Some authors suggested the use of FDG-PET/CT in monitoring malignant transformation of OPG in children with neurofibromatosis type 1 syndrome [17, 19]. Optic meningioma is a benign tumor that typically demonstrate minimal to no FDG uptake on PET [15] and can be associated with bony sclerosis/destruction as in our case (Fig. 3). Orbital pseudotumor could be both hyper or isometabolic on FDG PET [18]. Xie et al. described a 56-year-old female with elevated FDG activity in several cranial and peripheral nerves suggestive of multiple neuritis, with patient“s symptoms improving following treatment [3].

## Oculomotor, trochlear and abducens nerves (CN III, IV, VI)

**Fig. 4:**
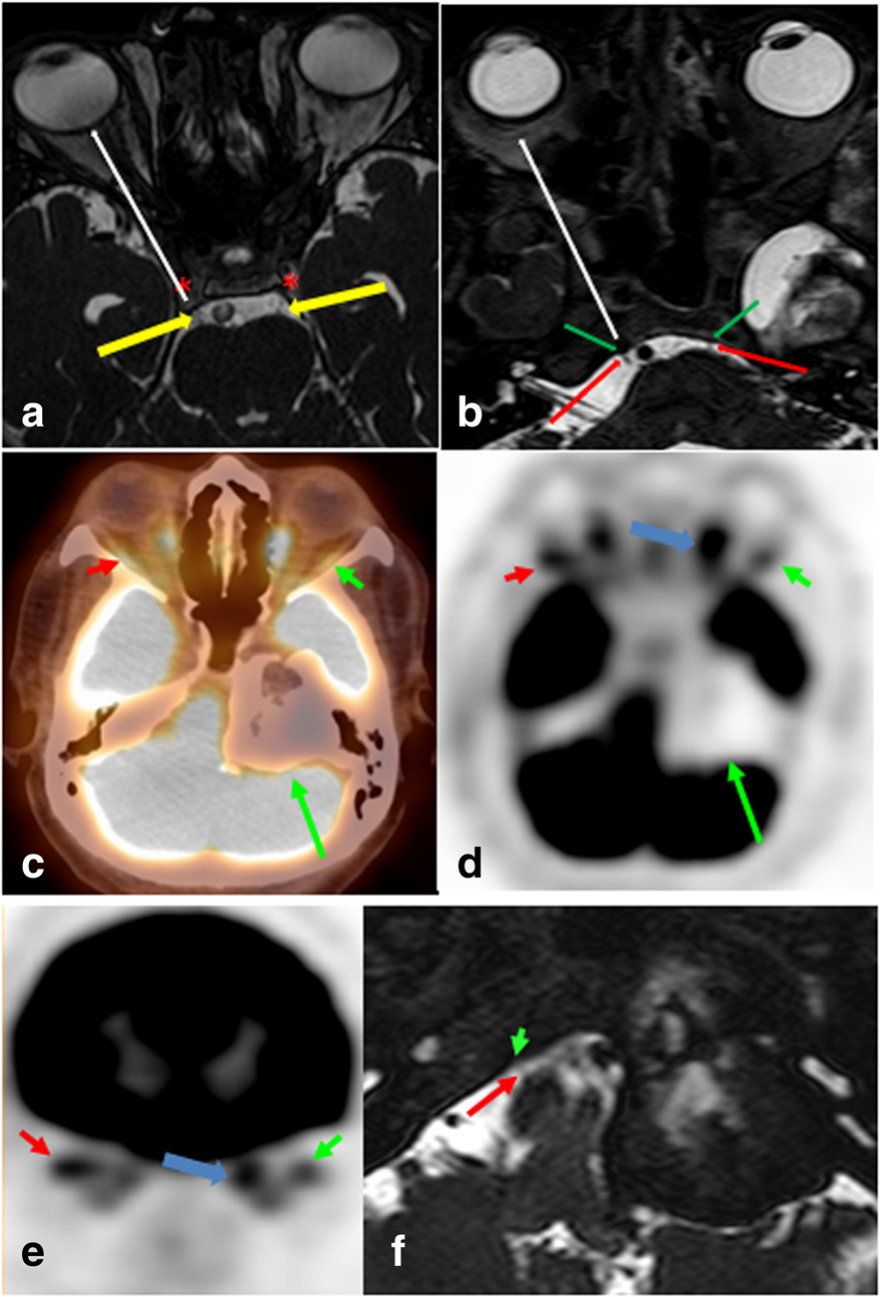
Axial Fast imaging employing steady-state acquisition (FIESTA) MRI image (**a**) showing the CNIII (yellow arrows) entering the cavernous sinus (asterix). From here, CNIII courses anteriorly in the dura of the cavernous sinus to enter the orbit through the superior orbital fissure (SOF), supplying the extra ocular muscles except the superior oblique and lateral rectus muscles. Axial FIESTA MRI image (**b**) shows the normal abducens nerves (red arrow) entering Dorello’s canal (green arrow). From here, CN VI courses anteriorly through the cavernous sinus to enter the orbit through the SOF, supplying the lateral rectus muscle. The white arrows show the general direction of travel of CN III and VI (**a**, **b**). A 73-year-old male presenting complaining of double vision. Axial fused PET/CT (**c**), PET (**d**), and coronal PET (**e**) images showing a large peripherally ossified hypometabolic expansile mass centered in the left petrous apex extending to the cerebellopontine angle and middle cranial fossa (long green arrow). Note the lower activity in the left lateral rectus muscle (green short arrow) compared to the right one (red arrow) and the overcompensating hypermetabolic left medial rectus muscle, indicative of left abducens nerve paresis which was confirmed clinically. **f** Zoomed in Axial FIESTA MRI imaging shows the normal right abducens nerve (red arrow) entering Dorello’s canal (green arrow). On the left side, the mass is seen involving the expected location of the left CN VI

Direct visualization of CNs III, IV and VI is usually beyond the resolution of PET/CT, however large brain stem or cavernous sinus lesions along the course of these nerves may indicate cranial nerve involvement by these lesions. Also, extraocular muscle atrophy or asymmetric decreased uptake could represent denervation injury, which should prompt a search for a lesion along the course of the innervating CN. In an attempt to compensate for the paralyzed muscle, the non affected extraocular muscles may show increased FDG activity, further confirming the CN involvement (Fig. 4c–e).

## Trigeminal nerve, maxillary and mandibular divisions (CNV, V2 & V3)

**Fig. 5:**
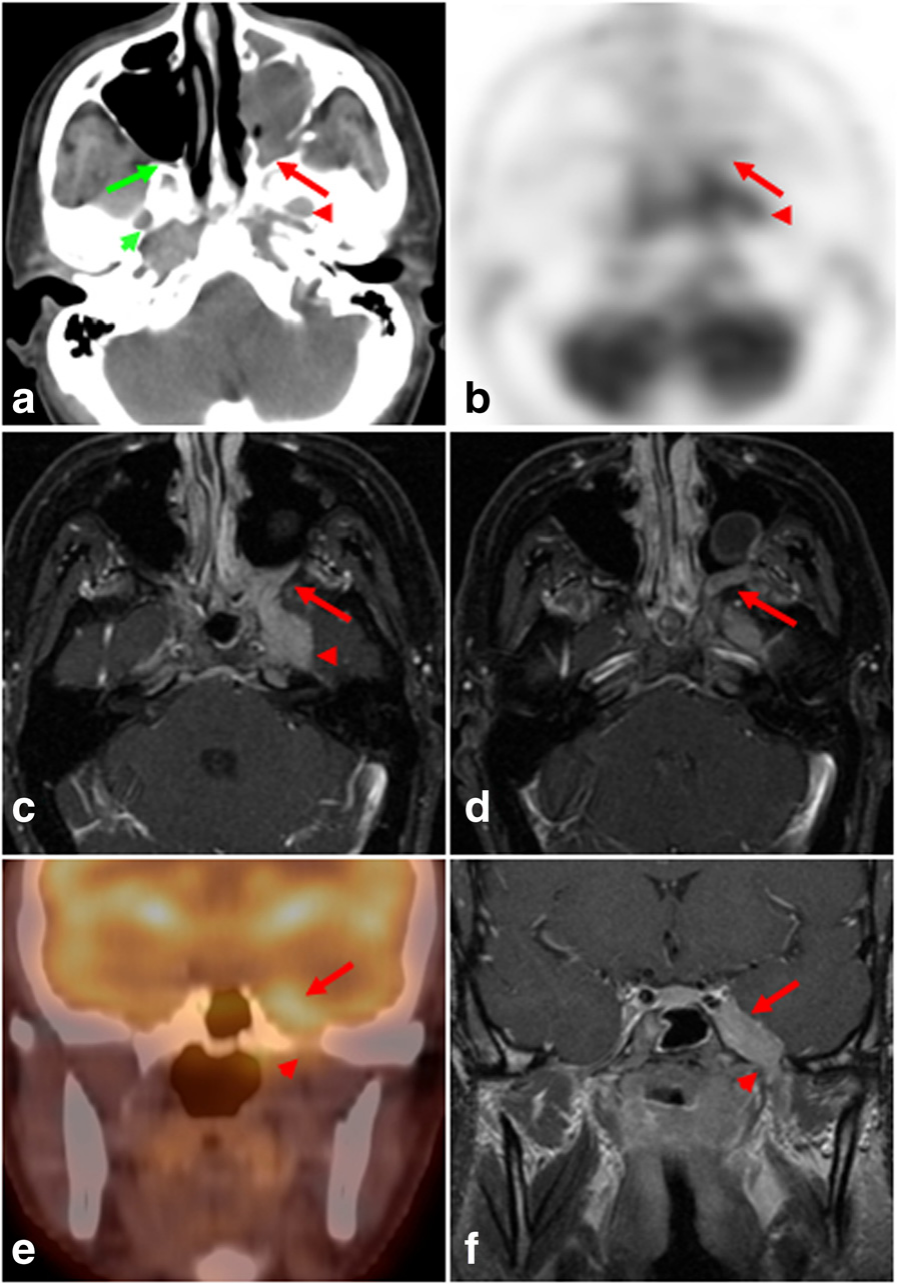
A 41-year-old male with nasopharyngeal squamous cell carcinoma. Axial CT (**a**), and PET (**b**) images show an iso/hyperdense hypermetbolic mass extending into the enlarged left pterygopalatine fossa (red arrow) and foramen ovale (red arrowhead), confirming perineural tumor spread along the maxillary (V2) and mandibular (V3) divisions of the trigeminal (CNV) nerve. Note the normal appearance of the right pterygopalatine fossa (green arrow) and foramen ovale (green arrowhead). Axial contrast-enhanced fat suppressed T1 SI MR (**c**, **d**) of the neck confirm extension of the enhancing soft tissue mass from the left cavernous sinus/Meckel’s cave region in to the left pterygopalatine fossa (red arrow). Coronal fused PET/CT (**e**) and contrast-enhanced fat suppressed T1 SI MR (**f**) confirming perineural extension of the tumor along V3, infiltrating the left cavernous sinus/ Meckel’s cave region (arrow) and widening the ipsilateral foramen ovale (arrowhead)

FDG-PET/CT can detect perineural tumor spread along the trigeminal nerve and its main divisions; most commonly arising from head and neck squamous cell carcinoma, adenoid cystic carcinoma, mucoepidermoid carcinoma, skin cancer and melanoma as well as [2] lymphoma [1] and neurolymphomatosis [20] (Fig. 5)

.

## Facial/ vestibulocochlear nerve complex (CNVII and VIII)

**Fig. 6:**
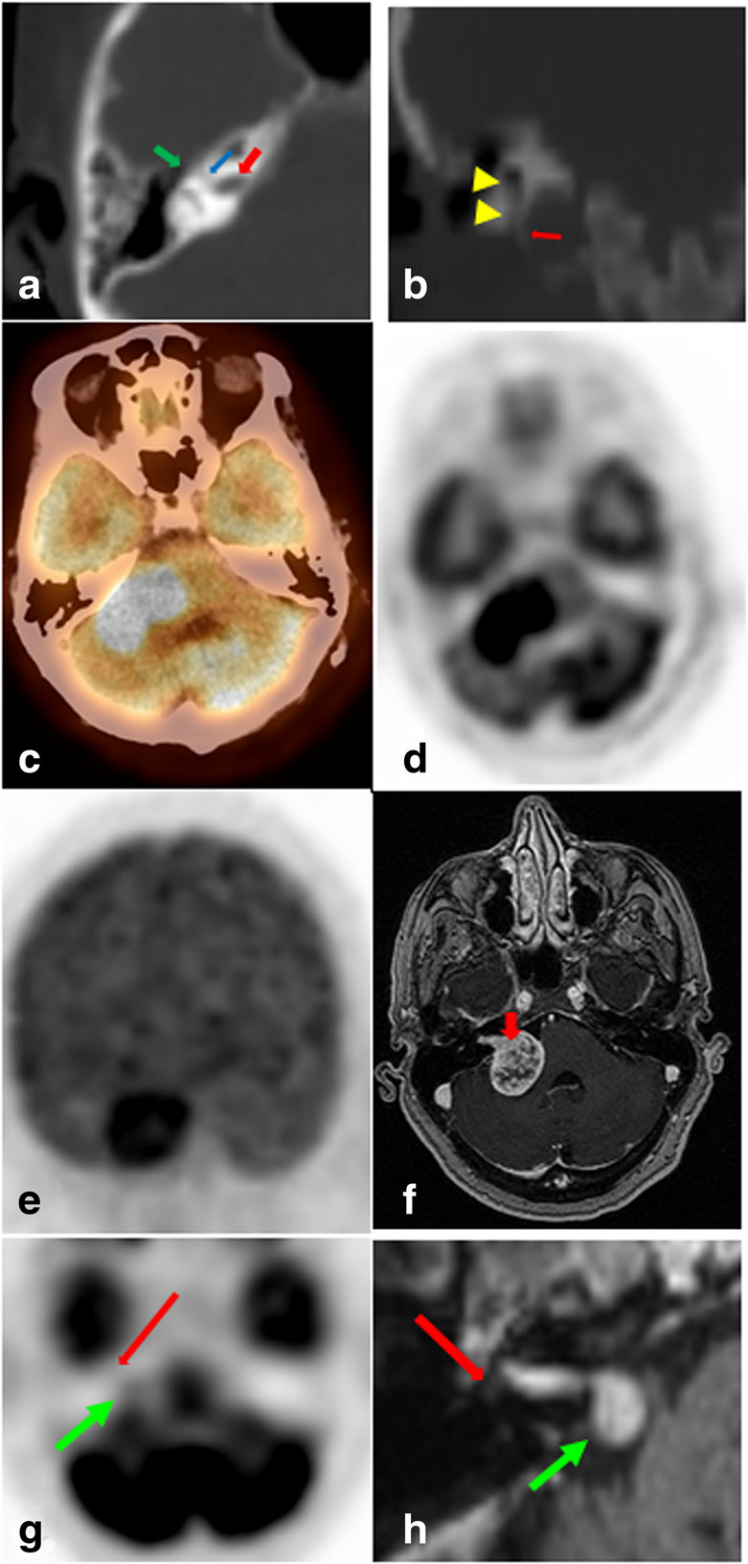
Axial (**a**) and coronal (**b**) low resolution CT part of PET/CT. **a** On the axial view the internal auditory canal parts of CNVII and VIII (red arrow), as well as the labyrinthine (bluearrow) and tympanic parts (green arrow) of CN VII can be visualized. **b** On the coronal view,the styloid process (red arrow) can be used as a landmark to visualize the stylomastoid foramen,containing the stylomastoid part of CN VII (arrow heads). **c**–**f** A 62 year-old-female with rightsided hearing loss. Axial fused/non fused FDG PET/CT (**c**, **d**) and coronal Maximum Intensity projection (MIP) (**e**) images showing an intensely hypermetabolic right cerebellopontine angle lesion (CPA) lesion. Given patient history of hearing loss, this was consistent with vestibular schwannoma, rather than a meningioma. Axial contrast enhanced T1 WI MRI (**f**) showing the classic heterogeneously enhancing “ice cream cone” mass extending into the right internal auditory canal (IAC) (arrow), consistent with vestibular schwannoma, which was confirmed pathologically. **g**, **h** A 71-yearold female patient with history of worsening right sided hearing loss, unsteadiness, and neck pain. Axial unfused PET/CT images (**g**) and contrast enhanced T1 WI MRI (**h**) show a right CPA enhancing lesion with mild focal FDG activity (SUVmax 3.0) (green arrow), extending along the IAC, labyrinthine and tympanic segments of CN7 (red arrow) confirming that this is a facial nerve schwannoma rather than the more common vestibular nerve schwannoma

The most common cerebellopontine angle lesions are vestibular schwannoma and meningioma. Vestibular schwannoma is typically described as a hypometabolic lesion [21], however in our experience they were hypermetabolic (Fig. 6c-f), which may be related to the large size of the lesions. Vestibular schwannoma is differentiated from meningioma by extension into the internal auditory canal (Fig. 6f). The less common facial nerve schwannoma is diagnosed when the lesion extends along the labyrinthine segment of CNVII (Fig. 6g, h). Perineural spread form parotid gland lesions should be suspected with abnormal activity extending superiorly along the stylomastoid foramen or within the temporal bone [2, 22, 23]. Rare CN melanoma metastasis along CNs VII and VIII has also been described [24].

## Vagus and spinal accessory nerves (CNs X and XI)

**Fig. 7:**
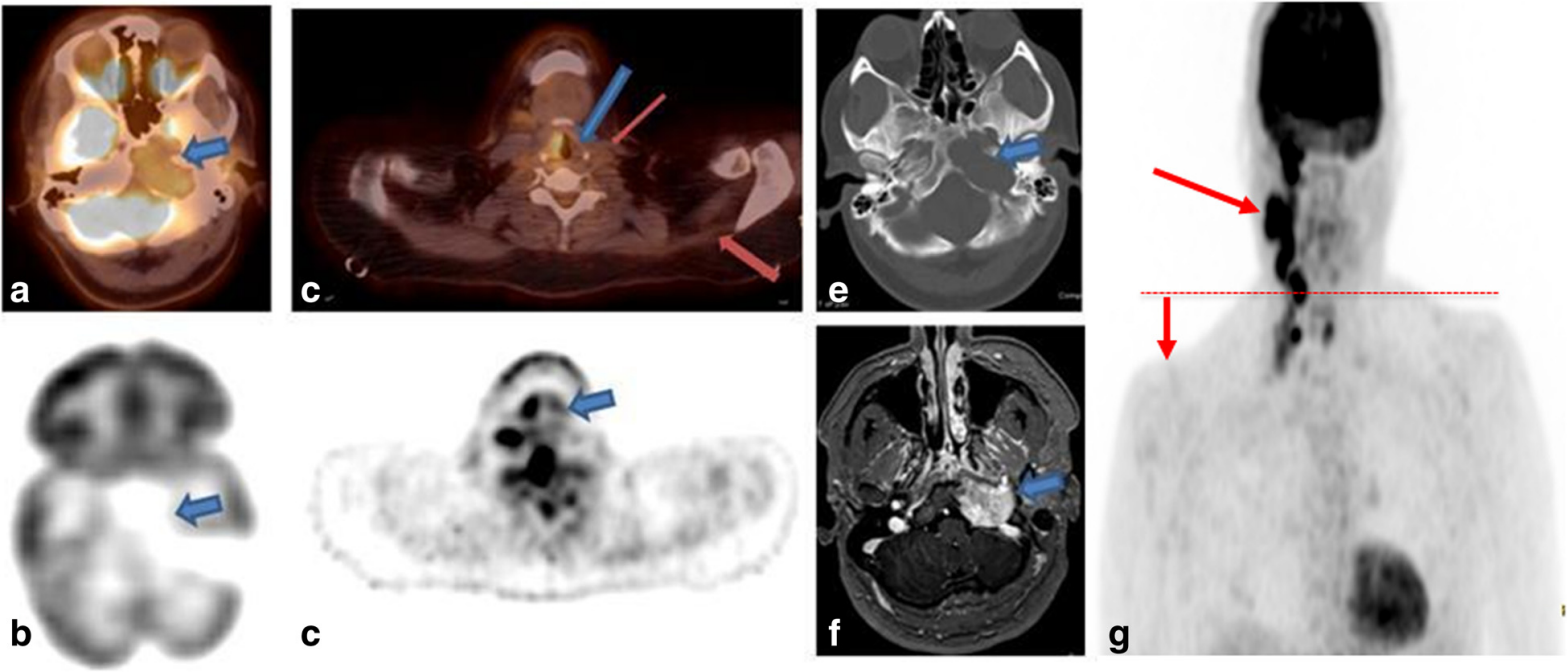
A 32 year-old-female presenting with a hoarse voice. Axial fused (**a**) and unfused (**b**) PET/CT images of the skull base showing a hypometabolic soft tissue mass centered on the left jugular foramen with smooth osseous expansion suggestive of schwannoma of cranial nerve IX, X or XI which all exit the skull base through the jugular foramen. Axial fused (**c**) and unfused (**d**) PET/CT images at the level of the glottis show no 18F-FDG uptake in the left vocal cord (blue arrow) with compensatory increased activity in the right vocal cord, consistent with laryngoscopy proven left vocal cord paralysis due to tumor involvement of left CN X and its recurrent laryngeal branch. Asymmetric atrophy of the left sternomastoid and trapezius muscles is consistent with chronic denervation due to tumor involvement of the left cranial nerve XI (spinal accessory nerve) (red arrows). Axial CT scan of the skull base with bone window settings (**e**) and axial post-contrast fat-suppressed T1 MRI (**f**) at the same level show the enhancing left cranial nerve IX/X/XI mass pathologically proven to be a schwannoma. **g** Coronal MIP image of a 50-year-old male with HIV presenting with worsening right facial weakness and pathologically proven squamous cell carcinoma of the neck, with perineural tumor invasion along the jugular foramen (not shown), showing right shoulder drooping compared to the left one (red line), secondary to atrophy of the right trapezius and sternomastoid muscles, confirmingCN XI involvement. Also note the bulky hypermetabolic cervical adenopathy (arrow) in the rightneck involving lymph node levels 1 through 4 consistent with metastatic lymph nodes

The most common jugular foramen (JF) lesions that my involve CN X and XI are glomus juglare, schwannoma, meningioma and skull base metastasis. Looking at the bone margins of the JF on the bone window of PET/CT may help differentiate glomus tumors which tend to have a permeative destructive margins from schwannoma which tend to cause smooth expansion of the JF (Fig. 7e) and meningioma, which may have permeative sclerotic margins [25]. If the recurrent laryngeal branch of CNX is involved, it will be seen as a hypometabolic ipsilateral paralyzed vocal cord with a hypermetabolic overcompensating contralateral vocal cord (Fig. 7c, d). Ipsilateral shoulder dropping on MIP images (Fig. 7g), with atrophy of the trapezius and sternomastoid muscles on the axial images (Fig. 7c, d), signifies CNXI involvement, which could be secondary to CNXI sacrifice during neck dissection.

## Hypoglossal nerve (CN XII)

**Fig. 8:**
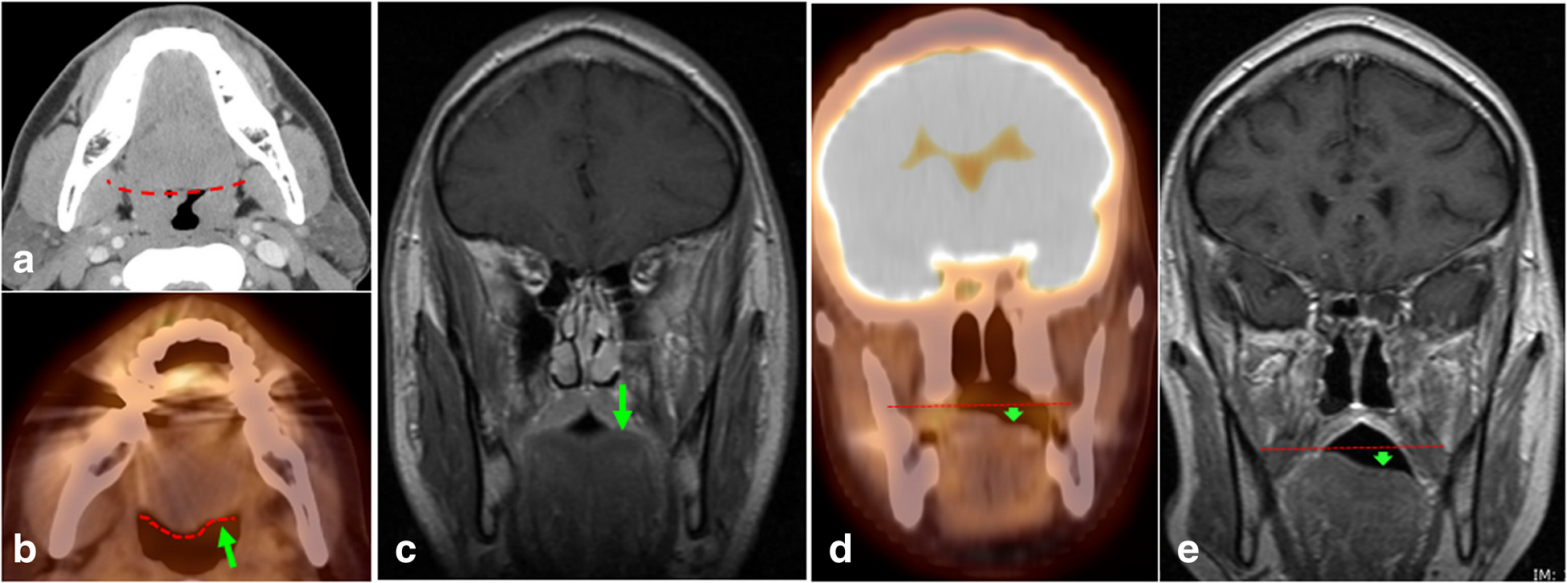
A 41 year-old-male patient diagnosed with left sided nasopharyngeal carcinoma and intracranial involvement. Axial CT of the neck (**a**) at the time of diagnosis (5/2005) showing normal posterior contour of the tongue (red line). The patient received radiotherapy and chemotherapy ending in 2006. Follow-up axial fused PET/CT images of the neck region (**b**) on (8/2013) shows interval atrophy of the left hemi-tongue, with abnormal posterior contour (green line). Post contrast coronal T1 WI before treatment (**c**), coronal fused PET/CT and coronal contrast enhanced MRI T1W images after treatment (**d**, **e**) confirming the left hemi tongue atrophy (arrow). Clinically, the patients tongue deviates to the left. The constellation of findings is consistent with post radiation hypoglossal neuropathy

Injury of CNXII could occur by the aforementioned JF lesions [25]. Further distally it could be secondary to hypoglossal foramen lesions (CNXII Schwannoma [25, 26]), clival tumor (chordoma, chondrosarcoma and plasmacytoma) [25], or rarely could be secondary to retrospective perineural tumor spread from tongue base tumor or radiation injury. An atrophic sagging fatty infiltrated ipsilateral tongue will be seen with hypometabolism on PET/CT (Fig. 8b, d, e) [25].

## Conclusion

Cranial nerve pathology can be detected on FDG PET/CT. With the increased reliance on PET/CT in patient staging and follow-up, PET/CT interpreters should familiarize themselves with these findings as it may change patient staging and management.

## Consent

“This retrospective study was approved by the Saint Louis University IRB board”.

## Abbreviations

FDG: [18 F]-2- deoxy-D-glucose
PET/CT: Positron emission tomography with computerized tomography
CN: Cranial nerve
HN: Head and neck
CPA: Cerebellopontine angle lesion
IAC: Internal auditory canal
JF: Jugular foramen
CN I: Olfactory nerve
CN II: Optic nerve
CN III: Oculomotor nerve
CN IV: Trochlear nerve
CN V: Trigeminal nerve
CN V1: Ophthalmic division of trigeminal nerve
CN V2: Maxillary division of trigeminal nerve
CN V3: Mandibular division of trigeminal nerve
CN VI: Abducens nerve
CN VII: Facial nerve
CN VIII: Vestibulocochlear nerve
CN IX: Glossopharyngeal nerve
CN X: Vagus nerve
CN XI: Spinal accessory nerve
CN XII: Hypoglossal nerve
FIESTA: Fast imaging employing steady-state acquisition
MPR: Multiplanar reformatted images
SSFP: Steady state free precession
STIR: Short tau inversion recovery
MIP: Maximum intensity projection
